# Speaking to a metronome reduces kinematic variability in typical speakers and people who stutter

**DOI:** 10.1371/journal.pone.0309612

**Published:** 2024-10-16

**Authors:** Charlotte E. E. Wiltshire, Gabriel J. Cler, Mark Chiew, Jana Freudenberger, Jennifer Chesters, Máiréad P. Healy, Philip Hoole, Kate E. Watkins

**Affiliations:** 1 Wellcome Centre for Integrative Neuroimaging, Department of Experimental Psychology, University of Oxford, Oxford, United Kingdom; 2 Department of Psychology, School of Human and Behavioural Sciences, Bangor University, Bangor, United Kingdom; 3 Institute for Phonetics and Speech Processing, Ludwig-Maximilian-University, Munich, Germany; 4 Department of Speech and Hearing Sciences, University of Washington, Seattle, Washington, United States of America; 5 Department of Medical Biophysics, University of Toronto, Toronto, Canada; 6 Physical Sciences, Sunnybrook Research Institute, Toronto, Canada; 7 Department of Psychology, University of Cambridge, Cambridge, United Kingdom; The University of Texas at Austin, UNITED STATES OF AMERICA

## Abstract

**Background:**

Several studies indicate that people who stutter show greater variability in speech movements than people who do not stutter, even when the speech produced is perceptibly fluent. Speaking to the beat of a metronome reliably increases fluency in people who stutter, regardless of the severity of stuttering.

**Objectives:**

Here, we aimed to test whether metronome-timed speech reduces articulatory variability.

**Method:**

We analysed vocal tract MRI data from 24 people who stutter and 16 controls. Participants repeated sentences with and without a metronome. Midsagittal images of the vocal tract from lips to larynx were reconstructed at 33.3 frames per second. Any utterances containing dysfluencies or non-speech movements (e.g. swallowing) were excluded. For each participant, we measured the variability of movements (coefficient of variation) from the alveolar, palatal and velar regions of the vocal tract.

**Results:**

People who stutter had more variability than control speakers when speaking without a metronome, which was then reduced to the same level as controls when speaking with the metronome. The velar region contained more variability than the alveolar and palatal regions, which were similar.

**Conclusions:**

These results demonstrate that kinematic variability during perceptibly fluent speech is increased in people who stutter compared with controls when repeating naturalistic sentences without any alteration or disruption to the speech. This extends our previous findings of greater variability in the movements of people who stutter when producing perceptibly fluent nonwords compared with controls. These results also show, that in addition to increasing fluency in people who stutter, metronome-timed speech also reduces articulatory variability to the same level as that seen in control speakers.

## Introduction

Developmental stuttering is characterised by unplanned interruptions to the flow of speech. Perceivable characteristics can include tense pauses, repetitions, and prolongations of sounds. These characteristics vary greatly between people and day-to-day within an individual. Despite the heterogeneity of stuttering, there are numerous ‘fluency-enhancing’ techniques that reliably reduce the likelihood of stuttering. Using these techniques can result in fluent speech for people who stutter regardless of the day-to-day amount of stuttering for the individual speaker. These fluency enhancers often use an external timing cue to aid speech, including metronome-timed speech, speaking in unison with another speaker, and singing.

This ‘rhythm effect’ can be explained at a neurobiological level by two neural loops that pass through the basal ganglia: an “external timing network” comprising the basal ganglia and supplementary motor area (SMA) and an “internal timing network” made up of the premotor cortex, cerebellum and basal ganglia [1]. Evidence for the dissociation between internal and external timing networks for movement comes from animal and patient studies. Recordings from neurons in macaque SMA showed a preference for internally generated movements (producing a predetermined sequence without a cue) whereas neurons in premotor cortex showed a preference for movement sequences that were cued externally (via lights) [2]. Patients with lesions involving the SMA and basal ganglia often have difficulties when producing internally generated movements but benefit substantially when moving or speaking with an external cue [3–5]. Similarly, people with Parkinson’s Disease have a loss of dopamine production in the basal ganglia motor loops (substantia nigra) and often have difficulties with limb (and sometimes speech) movements that are internally generated, but less so during externally cued movements [6–10]. Beyond stuttering, recent reviews describe the critical role of the basal-ganglia-thalamo-cerebellar loops in speech and non-speech rhythm perception, production, and sensori-motor integration [11–13].

Neurobiological explanations of stuttering propose that signalling within the SMA-basal ganglia loop is noisy, which leads to breakdown of the speech stream [1, 14]. This may be particularly important where prosodic stress is applied within the speech stream and at the initiation of speech movements during naturalistic speech [15–17]. The cerebellum-premotor-basal ganglia loop, on the other hand, may act to support fluent speech production when speaking with a predictable external cue [1]. Evidence from structural and functional brain imaging studies show differences in the basal ganglia in people who stutter [1, 18, 19]. The amount of activity within the substantia nigra during fluent speech has been shown to correlate with clinical measures of stuttering “severity” in people who stutter [20–22]. Structurally, the left putamen and left cortical speech areas were found to have higher iron levels in people who stutter, which may be indicative of elevated dopamine levels [23]. Dopamine is critically involved in regulating timing cues and the balance between inhibitory and excitatory signals in the basal ganglia-cortical networks. Together, there is consistent evidence that the basal ganglia motor loops are important in the cause of stuttering, and that there is a dissociation between how these loops control internally generated and externally cued (speech) movements.

Research into the neural basis of the “rhythm effect” in people who stutter is limited. However, in a recent study, fMRI was used to compare blood-oxygen-level-dependent (BOLD) responses when people who stutter read a passage with, and without, an imagined metronome [24]. The stuttering group had increased functional connectivity involving the cerebellum during externally paced reading compared with reading without an imagined metronome. The control group, on the other hand, did not show additional recruitment of the cerebellum in the metronome condition and instead had greater activity in the SMA. This suggests that people who stutter recruit the cerebellum during a metronome-timed speech condition as an “organic attempt at compensation” [24]. This lends support to the idea that fluency enhancement is achieved because of the engagement in the cerebellum-premotor-basal ganglia loop when an external cue, such as a metronome, is present.

The behavioural effects of metronome-timed speech in enhancing fluency in people who stutter are well established. However, to date, evidence for this effect is derived from acoustic or perceptual measures of speech and speech fluency. The speech motor control system comprises the brain (central nervous system) and the vocal tract (peripheral nervous system). Noisy signalling within or between neural areas during the planning and execution of speech [1, 19, 21, 25, 26] is thought to result in reduced control within the vocal tract (peripheral output). Less precise articulator control in the vocal tract can in turn lead to noisy and inconsistent feedback to the brain. Therefore, the effect of the metronome on the underlying neural control of speech, is likely to be reflected in articulatory behaviours. Given the aforementioned hypothesis of two distinct neural loops for internal and externally cued speech [1, 27], it would follow that for people who stutter, internally cued speech (i.e. no metronome) is characterised by noisier measures of speech motor control in the central and peripheral system, which should be lower during the externally cued condition (i.e. metronome).

Previous studies have used the degree of variability of speech movements, that is, how similar the speech movements are to each other over repeated utterances, as an index of the degree of control over the speech motor control system. Accordingly, higher articulatory variability in fluent speech has been reported in studies that record repeated utterances [28], vowel sounds [29], targeted jaw movements [30], simple and complex pseudowords [28, 31, 32] and simple sentences [33–35]. These studies, however, are either limited to measuring external articulators i.e. the lips and jaw and not the inaccessible internal articulators i.e. the tongue, velum and larynx, or require wired sensors to be attached to the articulators, which is uncomfortable for the speaker and results in altered somatosensory feedback. It is important to avoid altering perceptual feedback in people who stutter since this can enhance fluency [36]. These methods also measure discrete points along the vocal tract, rather than capture the entire vocal tract. Here, we use vocal tract MRI to record the entire vocal tract as someone is speaking. This can help researchers to study articulatory dynamics, such as the coordination and variability, of a range of articulators and study this in the context of the specific articulators that are critical to formation of the sound. Vocal tract MRI is a novel use of MRI that records images of the entire midline of the vocal tract, uninterrupted and at good temporal and spatial resolution without the need to attach anything to the articulators. It is therefore well suited to measure small kinematic differences in perceptibly fluent speech between people who stutter and controls.

The finding of higher variability in articulatory movements in people who stutter during fluent speech, though consistent, is difficult to interpret. Smith and colleagues (2010) proposed that greater variability is caused by ‘weaker’ speech motor control during the fluent of speech of people who stutter [28]. Specifically, they suggested that this increased variability leads to a greater likelihood of the “breakdown” of speech due to noisy signalling and feedback within the system, causing a stuttered moment. If that were the case, people with higher variability should show higher measures of stuttering”severity”(e.g. the index provided by the SSI-4 [37]). However, none of the aforementioned studies reported such a relationship. This is unlikely to be a satisfactory explanation for the cause of stuttered moments, therefore. The Motor Skills Hypothesis suggests that people who stutter lie at the lower end of speech motor “skill” continuum, are resistant to practice, and are prone to speech errors [38]. As with other skills, increased demands on the system, such as anxiety or complexity of the task, lead to greater instability and more errors, whereas a reduction in these demands, or supportive environments, such as fluency-enhancing conditions, should reduce instability and the likelihood of errors.

Therefore, while the consistent finding of high variability during fluent speech appears to be an important difference in people who stutter, the reason for higher variability and the relationship between variability and stuttered moments is unknown. One way to address this problem is to test whether articulatory variability is reduced when the likelihood of stuttering is reduced, for example, under fluency-enhancing conditions.

Here, we first aimed to extend our previous work using vocal tract MRI, which showed greater variability during nonword repetition in people who stutter compared with controls, by measuring variability during longer, naturalistic sentences. Secondly, we also aimed to extend our knowledge by investigating the effect of a well-known fluency enhancer—metronome-timed speech—on speech movement variability. In achieving these aims, this study can also demonstrate that that vocal tract MRI is a reliable method for recording small differences in kinematic control during fluent speech in a variety of experimental conditions (nonwords, sentences, with fluency-enhancing techniques).

Our hypotheses were as follows:

H1: People who stutter would have more articulator variability compared with control speakers during un-paced (i.e. no metronome) sentence repetition.
H2: People who stutter would show less articulator variability during metronome-timed speech compared with un-paced speech.

If we detect changes to the variability of speech articulation during fluent speech between the non-metronome and metronome conditions, this would first indicate that speaking with a fluency enhancer directly leads to a change in the peripheral speech motor control system, in line with theories and data from the neuroscience literature. Furthermore, if people who stutter speak with the same low levels as controls during metronome-timed speech would indicate that there are no limits or biomechanical constraints within the peripheral speech motor control system of people who stutter relative to that of control speakers, (i.e. people who stutter *can* produce fluent speech with the same low levels of variability as control participants). We leverage the advantages of vocal tract MRI to investigate speech movements non-invasively along the length of the vocal tract; the lips, tongue and velum.

## Method

### Participants

We scanned 27 people who stutter (PWS) and 20 control participants (CON). Data were collected from the same participants and session as our previous study [32], though four participants who stutter did not take part in the current task due to time constraints or technical difficulties. Data sets from five participants (2 PWS, 3 CON) were excluded due to technical difficulties with the scanner and audio set up. One dataset (PWS) was removed as there was an insufficient number of fluent utterances for analysis purposes (see analysis plan section) and one dataset (CON) was excluded due to the words being pronounced in different ways over the repetitions. This resulted in a sample of 24 adults who stutter and 16 controls. The groups were balanced for gender, age and years of education (S1 Table). All people who stutter had at least a score of 18, corresponding to “mild” on the Stuttering Severity Instrument-4 [37]. Participants reported normal or corrected-to-normal vision and normal hearing. No participants had taken part in speech therapy within the last six months. During the experiment, participants were asked not to actively use any of techniques that might help them to maintain fluency.

Participants were recruited to the study between 2017 and 2020. The University of Oxford Central University Research Ethics Committee (R52173/RE001) approved the study. Participants gave informed written consent to participate in the study, in accordance with the Declaration of Helsinki, and with the procedure approved by the committee.

### Experimental procedure

Prior to the experiment, the participants practised speaking the stimuli out loud. During scanning, for both the metronome and non-metronome condition, three sentences were repeated 10 times each in a random order (total 60 trials). The non-metronome condition was run before the metronome condition to avoid potential carry-over effects from hearing the rhythm of the metronome. For each trial, the sentence was displayed on the screen and participants read the sentence out loud. For the metronome condition (“Met”), participants heard a metronome (2Hz). For the no-metronome condition (“NoMet”), participants spoke at their natural speaking rate. Each trial lasted seven seconds. Each sentence contained a multisyllabic target word from Motor Speech Evaluation [39] (see Analysis Procedure).

### MRI acquisition

Data were obtained using a 3-T MRI system (Prisma, Siemens) with a 64-channel head and neck receive array. Midsagittal images of the vocal tract were acquired with in-plane spatial resolution of 2mm X 2mm using a radial FLASH sequence (echo time/repetition time– 1.4/2.5ms) with golden-angle sampling. Images were reconstructed at 33.3 frames per second using a second-order spatiotemporal total generalized variation constraint [40]. Audio was recorded using a noise-cancelling microphone (Optoacoustics, FOMRIII), which substantially reduced the sound of the MRI scanner in the recording. Participants wore noise-cancelling headphones linked to the same system, which safely cancels the scanner noise, allowing them to hear themselves more clearly. This also aids inspection of the acoustic signal for any perceptual disfluencies (see Analysis Procedure, below).

### Analysis procedure

The audio and reconstructed videos of the vocal tract were synchronized and inspected for any disfluencies or non-speech movements including yawning and swallowing. Speech was judged to be dysfluent if a rater perceived a disruption in the audio or movement (*i*.*e*. *audible repetitions or prolongations of speech sounds*, *or silent “blocks”*) when watching the vtMRI videos. If these dysfluencies or non-speech movements affected the production any part of the sentence, the trial was excluded. The audio was then segmented acoustically in Praat [41]. The target utterance was extracted from the sentence based on clear acoustic signals before and after the target word. The extracted utterances are shown in italics. These were: 1) the release of /ð/ and closure formation of /v/ in “Bob knew th*e impossibility o*f the task” 2) release of /d/ and closure formation of /p/ in “Mike carrie*d artillery u*p the hill” 3) release of /d/ and closure formation of /t/ in “Peter ha*d a catastrophe a*t work”. The same utterances were extracted for every participant.

**Fig 1:**
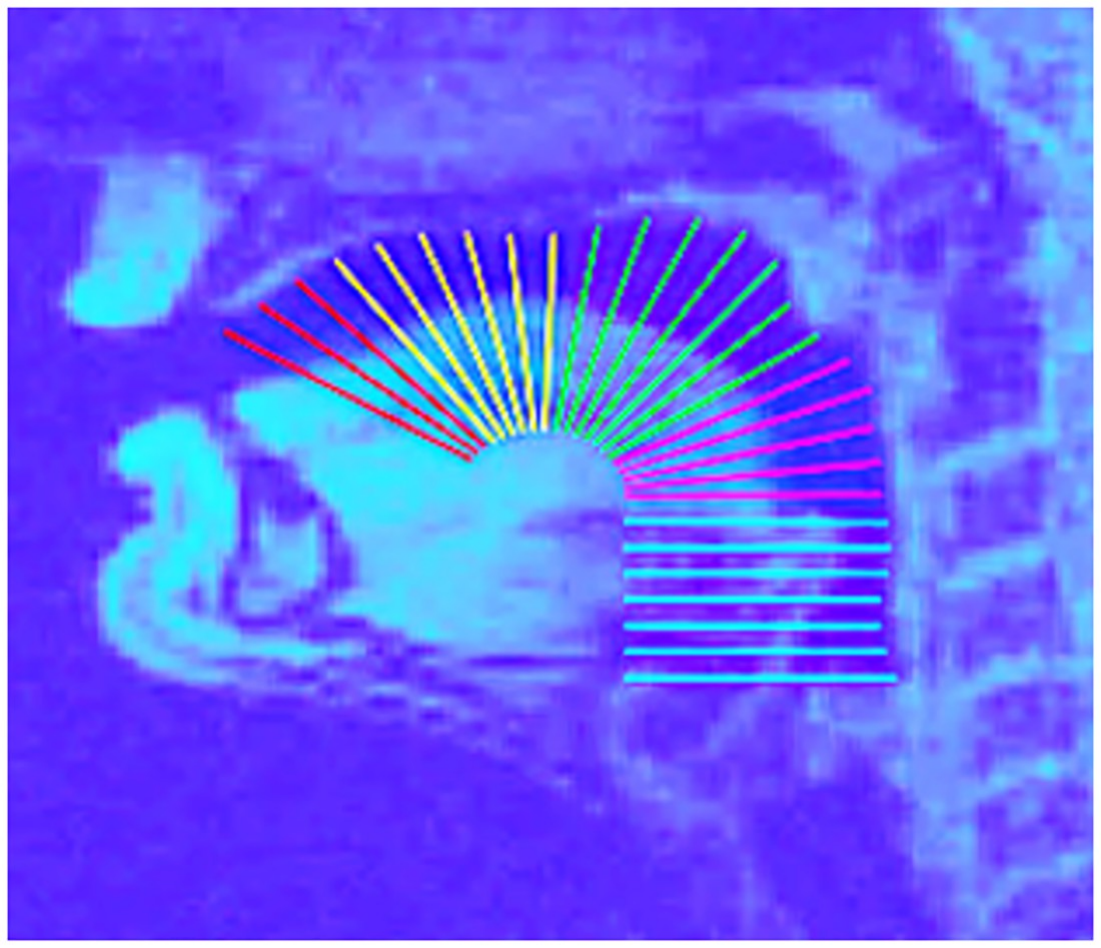
A frame with an open vocal tract (in this case, the schwa vowel in “the hill” during the metronome condition). Dark blue is air and bone, light blue is tissue. A semi-polar grid is constructed over the airway (see analysis procedures for details). Gridlines were coloured by region. Red = Alveolar, yellow = Palatal, green = Velar, pink = Hyperpharyngeal, blue = Hypopharyngeal.

The vocal tract images were segmented based on the manual acoustic segmentation. Vocal tract data were analysed using an open-source vocal tract MRI toolbox [42]. Whilst this toolbox is different from that used in our first vocal tract study [32], the analysis procedures are very similar. First, a frame with an open airway is chosen. Then, the glottis, velopharyngeal port, and alveolar ridge are marked manually. The midpoint of the line from the alveolar ridge to the glottis was located within the genioglossus muscle. This served as the origin for a semi-polar grid with 28 gridlines. The gridlines terminated at the automatically detected posterior or superior boundary. The mean pixel intensity is calculated for each gridline. The grid was then further divided into articulatory regions along the length of the vocal tract (See Fig 1). Therefore, the signal from each region is the mean of the mean pixel intensity for the relevant gridlines, scaled between zero and one for each participant. A large number of high-intensity pixels (i.e. tissue) along the gridline indicates high constriction of the vocal tract region. For the current analysis, we focussed on the alveolar, palatal and velar regions.

### Measured variables

**Fig 2:**
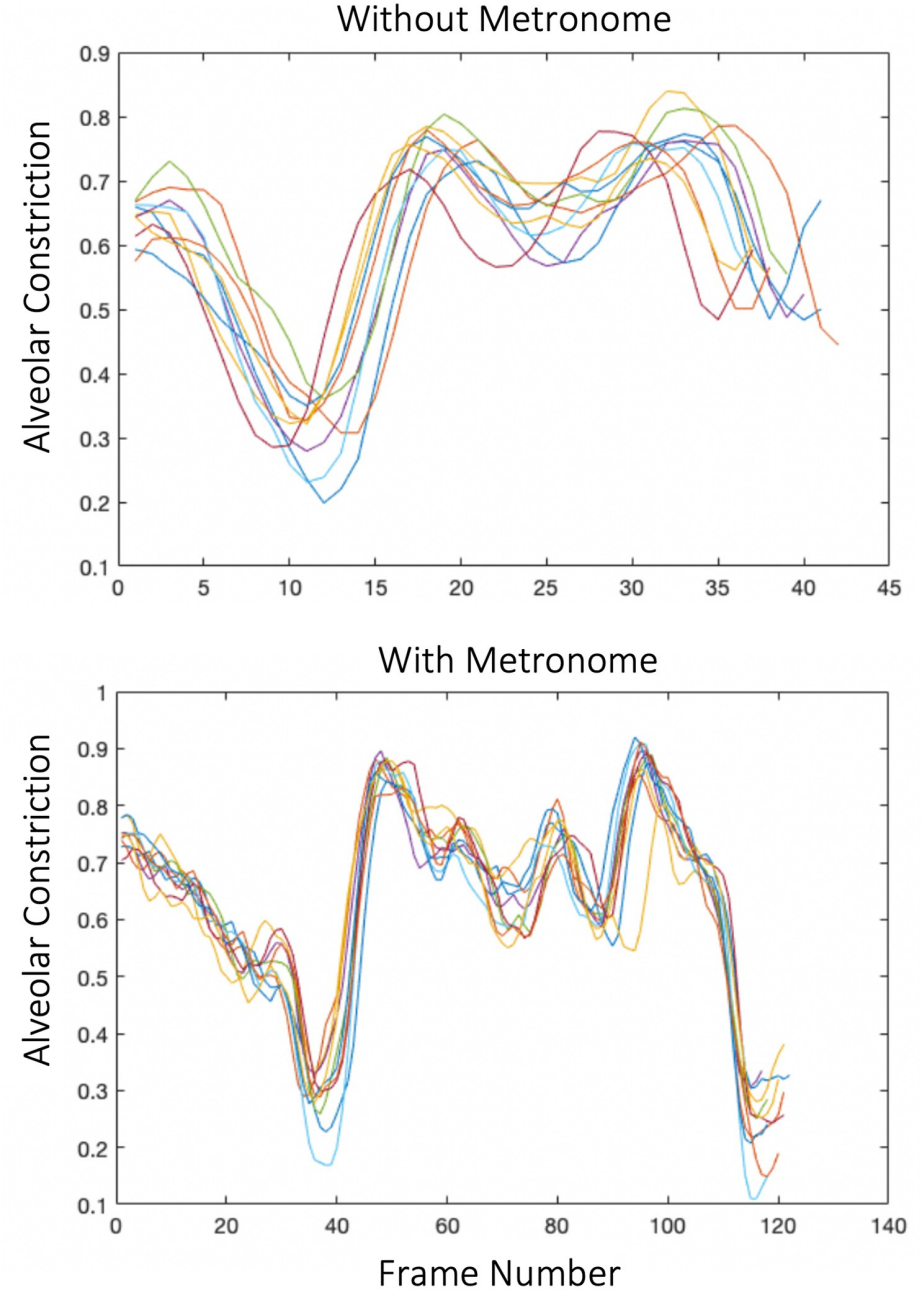
Example alveolar movement traces from one participant who stutters. Ten repetitions of the target utterance “*the impossibility of*” are overlaid (see “Analysis Procedure”, above). Each line represents one repetition. Data were reconstructed at 33.3 frames per second. Low constriction values mean the airway was more open.

Variability was calculated using the coefficient of variation (CoV), that is, the standard deviation of the size of the movements across 10 repetitions of each target utterance, divided by the mean. The size of the movement was simply the sum of the constriction value of the movements across frames capturing both the amplitude and duration of the movement (see Analysis Procedure and Fig 2).

Movement duration was recorded for each repetition by summing the total number of frames from the start to the end of the target utterance as defined above.

### Analysis plan

Trials resulting in exclusion were rare. If a participant did not produce at least six (out of 10) fluent and accurate productions of a sentence, data for that sentence were excluded from analyses. Trials containing stuttering were excluded here but will be used in future work [43]. Prior to analysis, two full data sets were removed; one from the stuttering group due to stuttering and one control participant due to mispronunciations. After removing these participants, a further 4.2% of sentences from the control group and 2% of sentences from the stuttering group were excluded (S2 Table). These exclusions were considered missing at random (that is, there was no systematic reason for exclusion) a necessary condition for statistical analysis.

## Results

**Fig 3:**
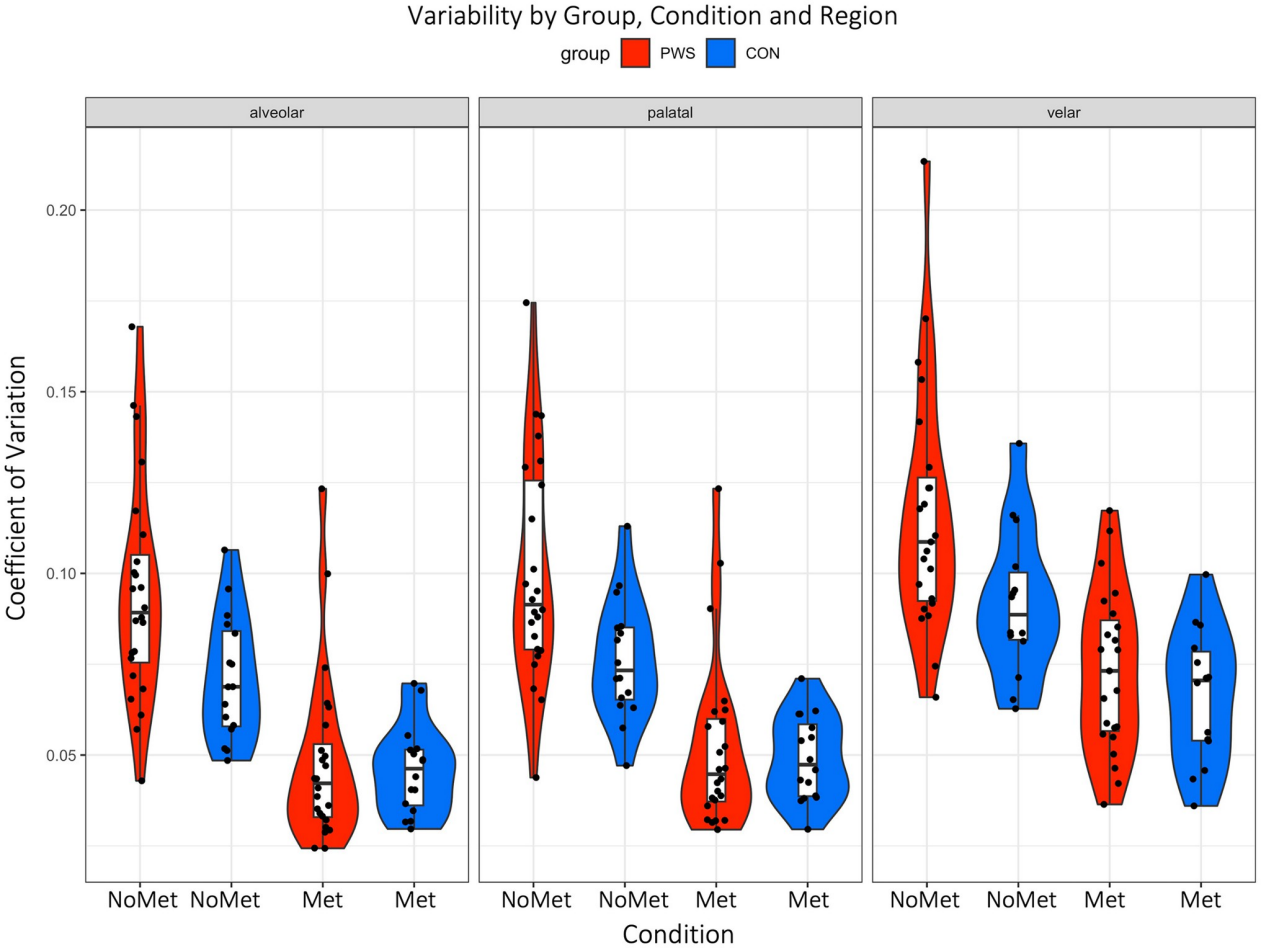
Coefficient of variation by group (PWS, CON), condition (“Met” and “NoMet”) and region (alveolar, palatal, velar). Each point is the variability of one participant averaged across the three sentences. Violin plots are shown to visualize the distribution of data and its probability density for each group and condition separately for each region of the vocal tract. Boxes display median and interquartile range; whiskers represent an additional 1.5*IQR.

**Fig 4:**
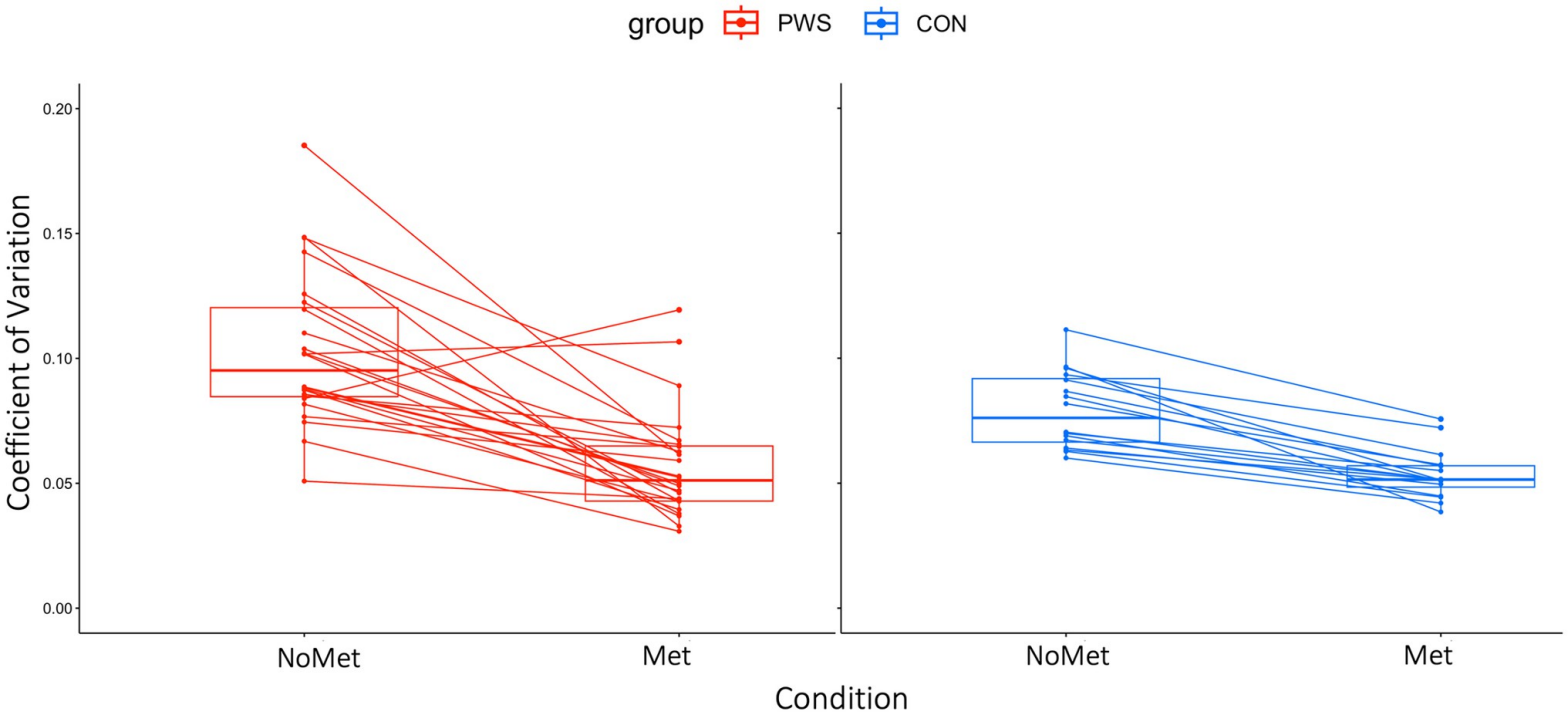
Interaction between condition (“Met” and “No Met”) and group. A line connects each participants’ data between the conditions. Data are averaged for each participant over region (alveolar, palatal, velar) and sentence. Boxplots display median and IQR, whiskers represent an additional 1.5*IQR.

**Fig 5:**
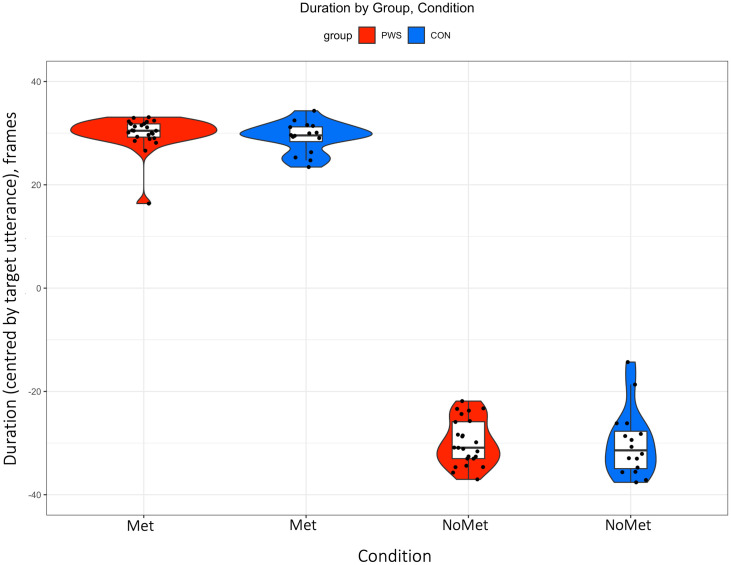
Average duration for each participant for the metronome (“Met”) and no metronome (“NoMet”) condition measured in frames (images reconstructed at 33 frames per second). The mean of duration was centred for each of the three target utterances prior to averaging to account for differences in utterance length between the three utterances. Violin plots are shown to visualize the distribution of data and its probability density for each group and condition separately. Boxplots display median and interquartile range; whiskers represent an additional 1.5*IQR.

Summary statistics are given in S2 Table. The amount of variability (CoV) for each condition (with or without metronome) and each region (alveolar, palatal and velar) is plotted in Fig 3. A summary variability score for each participant (collapsed across region) is plotted in Fig 4. Duration of the utterances for condition (“Met”, “NoMet”) are plotted in Fig 5.

We examined whether variability in speech movements during fluent repetitions of simple sentences differed 1) between people who stutter and a control group and 2) when speaking with, or without, a metronome. To do this, the *brms* package [44] was used to fit two Bayesian regression models. In the first model, we tested whether the coefficient of variation during the “NoMet” condition (i.e. natural speech) could be predicted by group (people who stutter vs. the control group) and region (alveolar, palatal and velar). In the second model, we tested whether coefficient of variation could be predicted by group, condition and region, as well as the interaction between group and condition. Region was not expected to interact with either of the other variables and therefore was not included as an interaction term. For both models, sentence was nested within participant as a random factor. Based on previous literature [32], mildly informative priors for the coefficient of variation were set between zero and 0.5, as the coefficient of variation cannot be negative and is unlikely to be above 0.5. This approach is not overly specific but allows for computational efficiency. A lognormal data distribution gave the best fit to the models. These models fitted well with *rhats* uniformly at 1, a large number of effective samples as well as stationary and well mixing chains (see R code for details of diagnostics).

### Differences in variability between groups

Variability (coefficient of variation) was predicted by group and region (Fig 3) using the first model. The stuttering group overtly produced the sentences with higher variability than the control group (β = 0.19; lower 95% credible interval (CI) = 0.02, upper 95% credible interval (CI) = 0.37). The velar region moved with more variability than both the alveolar (β = 0.24; CI = 0.19–0.21) and palatal regions (β = 0.17; CI = 0.12–0.22), which were similar (β = 0.07; CI = 0.02–0.12).

### The ’Metronome effect’ on variability

In the second model, the coefficient of variation was predicted by condition, with higher variability for the “NoMet” compared with the “Met” condition (β = 0.42; CI = 0.34–0.51) and an interaction between group and condition, with the stuttering group showing greater variability than controls in the “NoMet” condition, as expected based on previous work, but groups showing no difference in the “Met” condition (β = 0.20; CI = 0.09–0.31). As with the first model, velar movements were more variable compared with both alveolar and palatal movements, which were similar.

### Relationship between stuttering and variability

We selected the coefficient of variation for the “NoMet” condition, averaged across sentences and regions, as an overall measure of variability. As we had no *a priori* reason to select one region or one sentence as being more representative than another, we chose to average all the data to give a broad measure of variability from across the vocal tract. There was no correlation between variability score and SSI-4 score (*r* = 0.21, *p* = 0.33).

### Duration

The duration of the “Met” condition was considerably longer than the “NoMet” condition, as expected, given the pace of the metronome (2Hz). There were no differences in average duration or variance of the durations between the groups for the “NoMet” or “Met” conditions. As expected, given the predictable timing constraint of the metronome, the variance of the durations was larger for the “NoMet” condition compared with the “Met” condition for both groups.

## Discussion

### Summary of findings

We used vocal tract MRI to capture movements within the alveolar, palatal and velar regions at high temporal and spatial resolution in 24 people who stutter and 16 controls. We recorded images of the vocal tract as participants repeated simple sentences with and without a known “fluency-enhancer”, metronome-timed speech. First, we report that the stuttering group spoke with greater variability compared with the control group during natural speech that was perceived as fluent (i.e. without the metronome). Secondly, we show that both people who stutter and control speakers reduce their speech motor variability when speaking to a metronome, and that this effect is larger in the stuttering group. Overall, the variability of the stuttering group reduced to the same level as the control group when speaking to a metronome. Finally, we found no group differences in the duration of the utterances and no correlation between kinematic variability and clinical measures of stuttering “severity”.

### Greater variability in the fluent speech of people who stutter

The current work adds to a substantial body of work demonstrating that during speech that is perceived as fluent, people who stutter speak with higher amounts of articulator variability compared with control participants. This result, therefore, represents a robust finding in the literature that can be reproduced using different methods (e.g. Optotrak, vocal tract MRI), stimuli (sounds, nonwords, sentences) and analysis procedures (including coefficient of variation, as used here, and the spatiotemporal index [45]). Importantly, we demonstrate that vocal tract MRI is a robust and sensitive method for measuring articulator movement throughout the entire vocal tract without the need for intrusive sensors. It is unclear, however, why the stuttering group speak with higher variability and what the underlying mechanism is. To understand the factors that influence speech motor variability, we added the metronome condition to our experimental design, to understand whether variability can be altered by a known “fluency enhancer”.

### Speaking to a metronome reduces variability

During the metronome condition, there were no differences in variability between the groups. These results suggest that, given supportive conditions for fluent speech (i.e. with a “fluency enhancer”), people who stutter show no differences in speech motor control compared with people who do not stutter. This shows that there is nothing fundamentally restrictive about the speech motor control system in developmental stuttering.

Metronome-timed speech is thought to work by providing the speaker with an external, predictable timing cue. Our results show that both controls and people who stutter reduced the variability in their speech when speaking to the beat of a metronome. This is perhaps not surprising, given that our measure of variability, the coefficient of variation, captures variance in the duration of the utterance and that the metronome beat restricts the duration of the utterance. Instead, the effect of interest is that the stuttering group reduced the level of variability to that of controls when speaking to a metronome.

### No relationship between variability and stuttering “severity”

Our results clearly demonstrate that there was no relationship between a clinical measure of stuttering (SSI-4) and the amount of variability in fluent speech for the stuttering group. Furthermore, not everyone in the stuttering group has variability values outside of the range of the control group during the no-metronome condition. This replicates previous findings [28, 32] and suggests it is unlikely that the main mechanism behind stuttering is high levels of variability that lead to a breakdown of the speech stream, resulting in moments of stuttering. With this in mind, we discuss two opposing explanations for the role of kinematic variability in developmental stuttering.

### What does “more variability” mean for people who stutter?

Articulatory variability is commonly used to index speech motor control. Subtle differences in both terminology and interpretation of articulatory variability exist among different theories, however. The prevailing interpretation is that greater variability could indicate ‘weaker’ speech motor control during perceptibly fluent speech. That is, signalling between key speech motor control regions of the brain is disrupted in the brains of people who stutter and this disruption leads to instability in the kinematic output. This has been suggested to contribute to the disruption of speech motor control leading to a stuttered moment [28]. Similar interpretations propose that variability represents “unstable” speech motor control, which is more prone to breakdown due to increased demands on the system [38]. Another, alternative interpretation, is that greater variability could be the result of attempts to maintain fluency. That is, having a more flexible and adaptive speech motor control system may allow people who stutter to avoid stuttering. This notion would help to explain the lack of relationship between variability and measures of stuttering “severity”. Our results provide support for both interpretations. In each, the demands (e.g. socio-cogntive aspects of anticipating upcoming stuttering) on the speech motor control system are higher for people who stutter when speaking without a “fluency enhancer”. These increased demands lead to higher variability due either to weaker speech motor control or to an increased need for a flexible and adaptive system. Again, in both scenarios, demands on the speech motor system are reduced when the external cue acts as a scaffold, resulting in less variability. To tease apart these hypotheses, the relationship between cognitive flexibility, the use of techniques to reduce or control stuttering and kinematic variability should be investigated. A final possibility to be considered is that higher levels of variability within the stuttering group compared with the control group could be due to subtle imperceptible dysfluencies, which are eliminated when speaking to a metronome beat.

### Variability throughout the vocal tract

We divided the vocal tract into three sections; the alveolar, the palatal and velar regions. The alveolar and palatal regions produced similar results in both groups of speakers. The velar region, however, had higher variability scores compared with the alveolar and palatal region in both groups. At first sight, this result contrasts with that of a previous study using vocal tract MRI, which reported lower velum variability compared with labial and palatal regions [32]. This difference may be explained by differences in the analysis procedures between the two studies. In the prior study, the upper boundary of the vocal tract was used to measure movement of the velum itself, but in the current study, the pixel intensities from the gridlines within in the “velar” region were extracted. This latter approach therefore captures tongue back movement and the limted velum movement. In addition, the upper boundaries of the vocal tract are fixed for the alveolar and palatal regions but not for the velar region, which may contribute to more variability for measures based on gridlines in the velar region. Furthermore, the corpus for the present study contained very few sounds with strong constraints on the position of the tongue in the velar region.

### No group differences for the duration of utterances

There was no difference in duration between people who stutter and the control group during the no-metronome condition. There was also no difference during the metronome condition, however this was expected given the restricted timing provided by the metronome. Previous studies suggest that people who stutter slow down when productions become more complex [15, 38, 46, 47]. Slowing speech could be a compensatory mechanism (whether conscious or implicit) to maintain fluency when utterances are more difficult. Our previous study, which used vocal tract MRI to assess kinematic variability during pseudoword repetition reported that people who stutter slowed their speech as the pseudowords increased in complexity (both length and phonological complexity). In the current study, however, participants were producing simple, English sentences that they had seen prior to the vocal tract recordings. In contrast to our previous work therefore, these utterances may not have been difficult enough to warrant the use of slowed speech as a compensation strategy, thus we find no differences between the stuttering and control groups for the duration measure.

Our measure of kinematic variability is the coefficient of variation of the area under the curve of the movement trace for each articulator. This approach is sensitive to differences in both duration and movement amplitude. There was no group difference in the variability of duration, which suggests that the differences in kinematic variability are predominantly due to amplitude differences, rather than duration differences.

### Limitations

One key limitation of this work is that the metronome was slower than the participants’ natural speech rate and therefore it is difficult to separate the effects of the metronome and the effects of simply slowing speech. Stuttering frequency reduces at slower speech rates [15, 38] In addition, our previous findings [32] showed that people who stutter slowed their speech when productions were more phonologically complex. Taken together, this suggests that slowing speech is a useful mechanism for maintaining fluency for people who stutter and may contribute to the reduction in variability when speaking in the metronome condition. However, prior kinematic work found that adults who stutter had increased spatiotemporal index (spatial variability) when speaking at speeds that were half their natural rate [48]. This perhaps provides support that the reduction in variability found in the current work is related to the effect of the metronome, rather than just slowed speech. Further work that varies the speeds at which people speak under typical and fluency enhanced conditions would help to tease apart the effects of speech rate and variability.

The SSI is a tool that is commonly used to measure the severity of stuttering in individuals who stutter. However, the SSI only measures the severity of stuttering based on the frequency of stuttering events and the amount of visible tension there is during speech. Furthermore, it also only captures speech at one point in time which is particularly important given the high degree of day to day or moment to moment fluctuation that can occur in the fluency of speech for people who stutter. The lack of relationship between SSI score and variability score could be due to problems with the measurement tool. However, we believe this is unlikely given that previous work has also failed to find a correlation. It is more likely, in our opinion, that clinical measures of stuttering are not linked to speech motor variability.

### Future directions

Other populations of speakers with movement disorders, including people with Parkinson’s Disease, SMA syndrome and Tourette’s Syndrome also show fluency enhancing effects from external cueing techniques. As with stuttering, people with Parkinson’s disease or SMA syndrome experience more difficulties with movement initiation. Future work could investigate whether variability is greater at the initiation of perceptibly fluent speech compared with within-word transitions. Vocal tract MRI could be used with these other speaker populations to measure motor control or track therapeutic outcomes. We have demonstrated that speech motor variability can be captured non-invasively and quickly (10 minutes), making this approach suitable for these populations. Variability is also routinely measured in the general movement domain to look at whole body or limb movements. The resulting kinematic data is very similar to that produced by vocal tract MRI and as such, these multiple movement modalities could be compared directly, within participants, to understand the motor system as a whole. In addition, vocal tract MRI can be used to record instances of stuttering. We have demonstrated this approach with a case study [43], however, more systematic investigation of stuttering would be beneficial. This could be achieved using methods to elicit more stuttering during data collection [46, 47]. Coupling vocal tract imaging of stuttered speech with simultaneous acquisition of MRI-compatible techniques such as functional near infra-red spectroscopy (fNIRS) or electrophysiological brain measures is another exciting advance that could further our understanding of the proximal cause of stuttering.

## Supporting information

S1 Table Participant demographics. (DOCX)

S2 Table Summary statistics. (DOCX)
