# Supplementary material for: Speaking to a metronome reduces kinematic variability in typical speakers and people who stutter
Source: PLoS One. 2024 Oct 16;19(10):e0309612. doi: 10.1371/journal.pone.0309612 (PMC11482672; doi:10.1371/journal.pone.0309612)
Supplement: S1 Table — (DOCX) [file pone.0309612.s001.docx]

**Table S1:** Participant Demographics.

|  | **Stuttering (N=24)** | **Control (N=16)** |
| --- | --- | --- |
| **Gender** |  |  |
| men | 19 (79 %) | 13 (81 %) |
| women | 5 (21 %) | 3 (19 %) |
| **Age (Years)** |  |  |
| Median [min,max] | 33 [19,45] | 29 [19,44] |
| **Handedness** |  |  |
| left | 2 (8 %) | 1 (6 %) |
| right | 22 (92 %) | 15 (94 %) |
| **Education (Years)** |  |  |
| Median [min,max] | 16 [10,22] | 18 [13,24] |
| **Age of Stuttering Onset (Years)** |  |  |
| Median [min,max] | 5.0 [3.0,10] |  |
| Missing | 3 (12.5%) |  |
| **SSI Score** |  |  |
| Median [min,max] | 24 [18,36] |  |
| **Therapy (Years)** |  |  |
| Median [min,max] | 1.0 [0,14] |  |
| Missing | 1 (4.2%) |  |
| *Missing values represent information that participants could not recall.* | | |
