# Supplementary material for: Speaking to a metronome reduces kinematic variability in typical speakers and people who stutter
Source: PLoS One. 2024 Oct 16;19(10):e0309612. doi: 10.1371/journal.pone.0309612 (PMC11482672; doi:10.1371/journal.pone.0309612)
Supplement: S2 Table — (DOCX) [file pone.0309612.s002.docx]

**Table S2.** Summary Statistics.

| Group | Condition | region | % of samples | Summed Amplitude | CoV |  |
| --- | --- | --- | --- | --- | --- | --- |
| CON | Met | Alveolar | 100 | 53.892 | 0.045 |  |
| CON | Met | Palatal | 100 | 47.826 | 0.049 |  |
| CON | Met | Velar | 88 | 36.757 | 0.066 |  |
| CON | NoMet | Alveolar | 100 | 19.450 | 0.071 |  |
| CON | NoMet | Palatal | 100 | 17.719 | 0.076 |  |
| CON | NoMet | Velar | 88 | 13.496 | 0.091 |  |
| PWS | Met | Alveolar | 100 | 56.070 | 0.048 |  |
| PWS | Met | Palatal | 100 | 50.584 | 0.052 |  |
| PWS | Met | Velar | 96 | 34.830 | 0.073 |  |
| PWS | NoMet | Alveolar | 99 | 20.060 | 0.093 |  |
| PWS | NoMet | Palatal | 99 | 17.853 | 0.099 |  |
| PWS | NoMet | Velar | 94 | 12.991 | 0.115 |  |
| *“Met” = metronome condition, “NoMet” = no metronome (i.e. natural speech) condition. Missing participants for velar region due to failed articulator tracking; 2 CON, 1 PWS. Missing participants for one target utterance = 1 PWS. See “analysis plan” for details.* | | | | | | |
